# Quantitative disclosure of DNA knot chirality by high-resolution 2D-gel electrophoresis

**DOI:** 10.1093/nar/gkz015

**Published:** 2019-01-15

**Authors:** Antonio Valdés, Belén Martínez-García, Joana Segura, Sílvia Dyson, Ofelia Díaz-Ingelmo, Joaquim Roca

**Affiliations:** Molecular Biology Institute of Barcelona (IBMB), Spanish National Research Council (CSIC), Barcelona 08028, Spain

## Abstract

The characterization of knots formed in duplex DNA has proved useful to infer biophysical properties and the spatial trajectory of DNA, both in free solution and across its macromolecular interactions. Since knotting, like supercoiling, makes DNA molecules more compact, DNA knot probability and knot complexity can be assessed by the electrophoretic velocity of nicked DNA circles. However, the chirality of the DNA knots has to be determined by visualizing the sign of their DNA crossings by means of electron microscopy. This procedure, which requires purifying the knotted DNA molecules and coating them with protein, is semi-quantitative and it is impracticable in biological samples that contain little amount of knotted DNA forms. Here, we took advantage of an earlier observation that the two chiral forms of a trefoil knot acquire slightly different electrophoretic velocity when the DNA is supercoiled. We introduced a second gel dimension to reveal these chiral forms in DNA mixtures that are largely unknotted. The result is a high-resolution 2D-gel electrophoresis procedure that quantitatively discerns the fractions of positive- and negative-noded trefoil knots formed *in vitro* and *in vivo* systems. This development in DNA knot analysis may uncover valuable information toward disclosing the architecture of DNA ensembles.

## INTRODUCTION

The analysis of knots found in circular molecules of DNA provides precious information because knots are topological invariants that footprint the left- and right-handed turns of DNA in the tridimensional space. DNA knots can form during the topological closure of linear DNA molecules (1). They are also formed by recombinases that rearrange intramolecular DNA sequences (2) and by type-2 topoisomerases that pass intramolecular segments of duplex DNA through each other (3–5). The characterization of DNA knots produced *in vitro* in these manners has allowed determination of biophysical properties of DNA in solution and the trajectory of DNA when it interacts with multiprotein complexes (1,4,6–8). DNA knotting can occur *in vivo* by the same mechanisms and, not surprisingly, DNA knots have been found in viral particles (9–12), bacterial chromosomes (9,13–16) and more recently in eukaryotic chromatin (17). However, the detection and topological characterization of these knots is not exempt of technical limitations, which often preclude the extraction of their valuable information.

**Figure 1.**
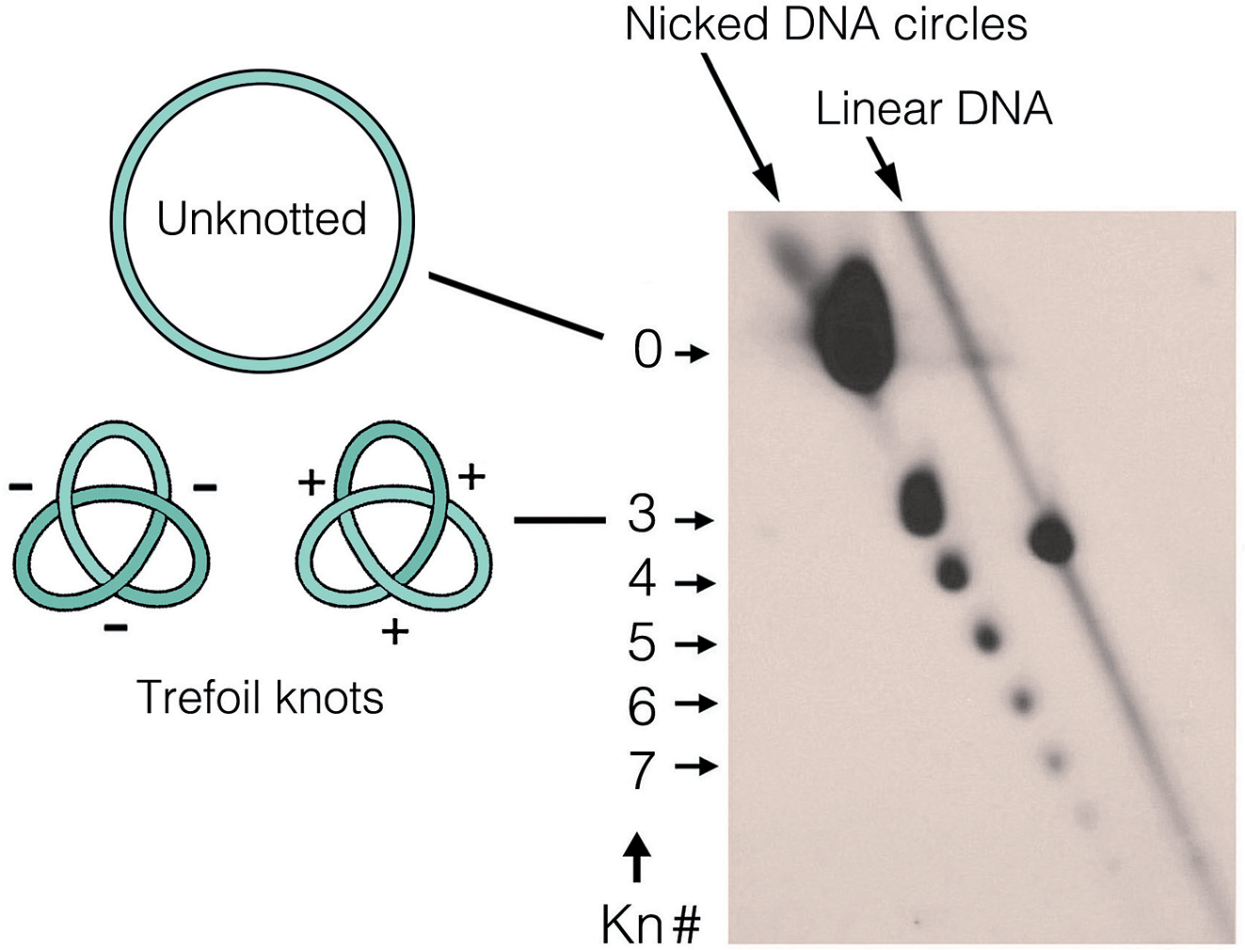
Electrophoretic analysis of DNA knot probability and complexity. The 2D gel-blot shows the spectrum of knots formed in YRp4, a 4.4-Kb DNA yeast circular minichromosome, *in vivo*. Upon nicking the DNA to abolish the molecular compaction caused by supercoiling, DNA was electrophoresed in 0.9% agarose gel in TBE buffer (89 mM Tris-borate, pH 8.3, 2 mM EDTA). The first gel dimension (top to bottom) was at 33 V for 42 h and the second (left to right) was at 150 V for 3 h. In these conditions, the velocity of DNA molecules containing a knot correlates to the knot complexity (Kn#, the irreducible number of DNA crossings in a knot). The gel signals of unknotted circles (Kn# 0), knots of Kn# 3 to 7 and linear DNA fragments are indicated. Note that nicked DNA circles containing positive- or negative-noded trefoils (kn# 3) have identical compaction and, therefore cannot be distinguished.

The probability of DNA knot formation and the complexity of a DNA knot (i.e. the number of irreducible DNA crossings in a knot) can be quantitatively assessed by agarose gel electrophoresis (18–20). This assessment is possible because the gel mobility of a duplex DNA circle is determined by its molecular compaction. Therefore, upon nicking of the duplex DNA to eliminate the compaction caused by supercoiling, DNA molecules containing a knot remain more compact. Consequently, knotted molecules move faster than the unknotted ones and their velocity correlates to the knot complexity (18–20). Accordingly, the simplest and slowest knot form is the trefoil (knot 3_1_), which has three irreducible DNA crossings. However, as the two mirror images of any knot produce the same compaction, it is impossible to discern through the electrophoretic velocity of nicked DNA circles the chirality of a knot (i.e. the trefoil of three positive crossings from that of three negative crossings) (Figure 1). To date, there are two approaches to unveil DNA knot chirality. The first is the currently used method of visualizing the topological sign of the irreducible DNA crossings of a knot by means of electron microscopy (EM). This procedure requires purifying the knotted DNA molecules and coating them with Rec-A protein to distinguish the over- and under-passing segments of DNA in each crossover (21). Semi-quantitative analysis of DNA knot chirality is attained by inspecting a large number of knotted molecules. The second approach is based on the former study of Shaw and Wang (22), who uncovered that positive- and negative-noded trefoil knots acquire slightly different electrophoretic velocity when DNA molecules are supercoiled, instead of nicked. This approach requires the purification of nicked DNA trefoils and subsequent introduction of DNA supercoils prior to electrophoresis. Therefore, as in the case of EM, this method is unfeasible in most biological samples, where the fraction of knotted DNA molecules is generally too small to be purified and processed.

Here we improved the electrophoresis method of Shaw and Wang (22) by introducing a second gel dimension that separates knotted DNA molecules from unknotted circles and linear fragments. The result is a high-resolution 2D gel electrophoresis that quantitatively discerns the chirality of DNA trefoils without having to purify them. We validate the method by showing the amount of positively and negatively noded trefoil knots produced *in vitro* upon circularization of linear DNA molecules, and upon incubation of supercoiled DNA with topoisomerase II. We apply next the method to uncover the chirality of the small fraction of trefoil knots that occur in bacterial plasmids and yeast circular minichromosomes *in vivo*.

## MATERIALS AND METHODS

### Circularization of free DNA

A total of 2 μg of YRp4 plasmid (17) was linearized by digestion with *Hin*dIII endonuclease (NEB) and the resulting 4.4-Kb DNA fragments were gel purified. A total of 1 μg of the linear DNA molecules were circularized by treatment with phage T4 DNA ligase (NEB) at 16°C during 1 h in 0.2 ml reaction mixtures containing 10 mM Tris–HCl (pH 7.5), 0.1 mg/ml bovine serum albumin, 10 mM 2-mercaptoethanol, 1 mM ATP, 4% (vol/vol) glycerol and 10 mM MgCl_2_. The reaction mixtures were phenol extracted twice and the DNA ethanol precipitated.

### Topoisomerase II-mediated knotting of DNA

A total of 1 μg of *Saccharomyces cerevisiae* purified DNA topoisomerase II (23), was mixed with 1 μg of negatively supercoiled plasmid (pUC19 or YRp4) with in a 0.1 ml volume containing 10 mM Tris–HCl (pH 7.5), 0.1 mg/ml bovine serum albumin, 10 mM 2-mercaptoethanol, 4% (vol/vol) glycerol, 20 mM KCl, 20 mM MgCl_2_and 2 mM spermidine. AMPPNP (Merck) was added to 2 mM and the mixture incubated at 30°C during 5 min. Reactions were terminated by the addition of sodium dodecyl sulphate to 1% and proteinase K to 0.1 μg/ml, and incubation at 60°C for 1 h. Reaction mixtures were phenol extracted twice and the DNA ethanol precipitated.

### Adjustment of DNA supercoiling density

DNA at a final concentration of 100 μg/ml was incubated with topoisomerase I (type-1B) of vaccinia virus (24) at 25°C during 15 min in DNA relaxation buffer (50 mM Tris–HCI, 100 mM NaCl, 8 mM MgCl_2_, **1** mM ethylenediaminetetraacetic acid (EDTA), **7** mM 2-mercaptoethanol) containing 250 μg/ml of chloroquine diphosphate (Merck). The same DNA products were obtained by nicking the DNA with Nt-*Bst*NBI endonuclease (NEB) and sealing the nicks afterward with phage T4 DNA ligase (NEB) in presence of 250 μg/ml of chloroquine. The reaction mixtures were phenol extracted three times and the DNA ethanol precipitated.

### DNA extraction from *E. coli* and *S. cerevisiae*

Liquid cultures of *Escherichia coli* HB101 cells transformed with YRp4 were harvested during exponential growth at 37°C and plasmid DNA extracted using the alkaline lysis method (25). Alternatively, plasmid DNA was extracted and purified with commercially available extraction kits (GenElute™ from Sigma). Liquid cultures of *S. cerevisiae* (strain FY251) transformed with YRp4 were grown at 26°C in yeast synthetic media containing 2% glucose. Before harvesting the cells during exponential growth, the DNA topology of circular minichromosomes was fixed *in vivo* by quickly mixing the cultures with one volume (−20°C) of ETol solution (95% ethanol, 28 mM toluene, 20 mM Tris HCl [pH 8.8], and 5 mM EDTA). Yeast cells were disrupted with glass-beads and DNA extracted as previously described (26).

### Analysis of knotted DNA by typical electrophoresis

**Figure 2.**
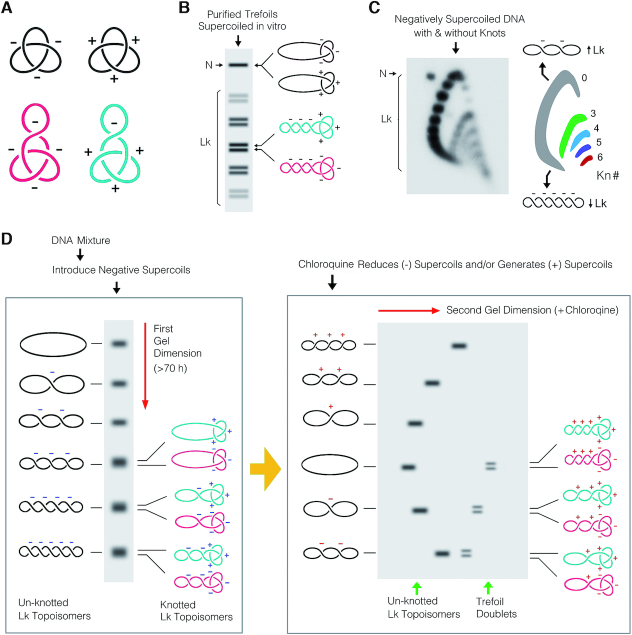
Electrophoretic separation of supercoiled DNA molecules that contain knots. (**A**) Unlike nicked DNA trefoils (black), the 3D contours of positive- and negative-noded trefoils (turquoise and purple) are no longer mirror images when the DNA molecules have supercoils. (**B**) Scheme of a 1D-gel illustrating the mobility of purified DNA trefoils, in which negatively supercoils had been introduced during nick sealing. After a prolonged electrophoresis, Lk topoisomers (Lk) of DNA trefoils split into doublets that correspond to positive- (top band) and negative-noded (bottom band) forms of the knot. N, nicked molecules (**C**) Separation of the Lk distributions of unknotted and knotted DNA molecules by 2D-gel electrophoresis. Negatively supercoiled pUC19 plasmid was knotted *in vitro* with a molar excess of topoisomerase II. Electrophoresis was done in 1% agarose gel in TBE (89 mM Tris-borate, pH 8.3, 2 mM EDTA) plus 0.4 and 2 μg/ml of chloroquine diphosphate respectively in the first (top to bottom, 60 V, 16 h) and second gel dimension (left to right, 60 V, 8 h). Lk distributions (arcs) containing knots of different complexity (Kn# 0, 3, 4, 5, 6) are colored. DNA becomes increasingly negatively supercoiled in an anticlockwise direction around the arcs. (**D**) Scheme of the high-resolution 2D-electrophoresis proposed to uncover the chirality of DNA trefoils present in complex DNA mixtures. Left panel: after introducing negative supercoils in the DNA, a long first gel-dimension resolves the Lk topoisomers of the two chiral forms of the DNA trefoils into doublets (turquoise and purple). However, these doublets are masked by the signals of more abundant DNA forms, mainly Lk topoisomers of the unknotted circle (black). Right panel: a second gel-dimension containing appropriate concentration of chloroquine alters differently the molecular compaction and thus the gel mobility of unknotted (black) and knotted Lk topoisomers (turquoise and purple). The doublets of trefoil signals then become visible for quantitative analysis.

For the electrophoretic analysis of DNA knot probability and complexity shown in Figure 1A, DNA was extracted from yeast cells containing YRp4 and incubated with the DNA nicking endonuclease BstNBI. The nicked DNA sample was analyzed by agarose 2D-gel electrophoresis as detailed in the figure legend. For the electrophoretic separation of supercoiled topoisomers of unknotted and knotted DNA shown in Figure 2C, pUC19 plasmid (2.7-Kb) was knotted *in vitro* with topoisomerase II and analyzed by agarose 2D-gel electrophoresis in presence of chloroquine as detailed in the figure legend. In both cases, 2D-gels were blot-transferred to nylon membranes, probed with DNA labeled with AlkPhos Direct (GE Healthcare^®^).

### High-resolution 2D-gel electrophoresis

A horizontal electrophoresis system supporting agarose gel sizes about 40 cm in length was used (for instance, System A3-1 of Owl™ for 23 × 40 cm gels). Gel slabs were cast with general purpose agarose for nucleic acid electrophoresis. Agarose from different suppliers (Molecular Biology grade Sigma-Aldrich^®^, Certified™ Molecular Biology Agarose BioRad^®^. Ultrapure grade agarose NYZTech^®^) yielded comparable resolution. For DNA molecules of about 5 Kb, a 0.9% agarose gel was cast in TBE buffer (89 mM Tris-borate, pH 8.3, 2 mM EDTA), containing appropriate concentration of chloroquine diphosphate (27). Chloroquine was omitted when the negative supercoiling density of DNA circles was adjusted prior to electrophoresis. Otherwise, the chloroquine concentration in TBE during gel casting and the first dimension of the electrophoresis was adjusted between 1 and 1.5 μg/ml for DNA samples extracted from eukaryotic and bacterial cells, respectively. The first gel dimension was run at room temperature (25°C) at 80 V for 70 h, without needing to replace, recirculate and control the buffer temperature during electrophoresis. Afterward, the gel was cut in two and the slab of about 23 × 23 cm corresponding to the bottom half gel was equilibrated during 1 h at room temperature with the second dimension TBE buffer containing 5 to 8 μg/ml chloroquine (about five-times the amount used in the first gel dimension), or 0.65 μg/ml chloroquine, when the first gel dimension had no intercalator. The gel slab was then electrophoresed in the orthogonal direction at room temperature at 120 V for 6–10 h. The gel was blot-transferred to a nylon membrane and probed with DNA sequences labeled with ^32^P or with AlkPhos Direct (GE Healthcare^®^). DNA signals obtained with increasing exposure periods were recorded on X-ray films or by phosphorimaging for quantitative analysis. Horizontal levelers were used during gel casting and during electrophoresis to optimize the electrophoretic resolution. Likewise, freshly made solutions and hybridization probes of high specific activity were used to minimize background signals that could mask the faint bands of knotted *Lk* topoisomers.

## RESULTS AND DISCUSSION

### Rationale of the procedure

This method combines two features of the interplay between DNA supercoiling and knotting on DNA velocity during gel electrophoresis. The first feature is that the spatial conformations of the two chiral forms of a DNA knot are no longer identical mirror images when the DNA molecule contain supercoils (Figure 2A). In this regard, Shaw and Wang demonstrated that, upon introduction of negative supercoils in gel-purified samples of DNA trefoils, negative-noded trefoils move slightly faster than the positive-noded ones (22). Consequently, after prolonged gel electrophoresis, the linking number (Lk) topoisomers of supercoiled DNA molecules containing a trefoil can split into doublets that correspond to the two chiral forms of the trefoil knot (Figure 2B). The second feature is that Lk topoisomers of negatively supercoiled DNA molecules that are unknotted can be separated from those Lk topoisomers that contain a knot by running a 2D gel in the presence of a DNA intercalator (i.e. chloroquine) (5). Increasing the concentration of chloroquine in the second gel dimension produces larger DNA unwinding, which neutralizes the molecular compaction produced by supercoiling, but not that caused by DNA knotting (27). As a result, Lk topoisomers of unknotted and knotted DNA molecules that had similar compaction and gel position during the first gel dimension acquire different velocity during the second dimension. Lk topoisomers form then a series of arcs in the 2D-gel, each corresponding to molecules of distinct knot complexity (Figure 2C). Therefore, in these conditions, there is no need to nick the DNA to uncover the presence of knotted molecules in DNA samples that are mostly unknotted.

On the basis of above premises, we hypothesized that combining a long first gel dimension, to separate the two chiral forms of the trefoil knot in negatively supercoiled DNA molecules, with a second gel dimension, to separate the knots from overlapping signals of unknotted molecules and linear DNA fragments, should expose the signals of positive- and negative-nodded trefoils for quantitative analysis (Figure 2D).

### Setting of DNA superhelicity and velocity during electrophoresis

To expose and achieve the desired electrophoretic resolution of DNA trefoils in complex samples, the superhelicity of DNA needs to be adjusted during the first and second gel dimensions of the electrophoresis. In the first dimension, the Lk distribution of unknotted DNA topoisomers should be resolved as a broad ladder spanning from relaxed to moderately negative supercoiled forms (Figure 2D, left). This degree of superhelicity can be attained in two manners. When the DNA molecules in the sample are negatively supercoiled, a suitable separation of the Lk topoisomers is achieved by running the first gel dimension in the presence of an appropriate concentration of chloroquine. For DNA molecules extracted from bacterial and eukaryotic cells, this concentration is between 1 and 1.5 μg/ml. Alternatively, when the DNA molecules in the sample are not negatively supercoiled (i.e. relaxed or positively supercoiled), the adequate DNA superhelicity is generated prior to electrophoresis by incubating the DNA sample with topoisomerase I (type-1B) in the presence of 250 μg/ml of chloroquine. The first gel dimension is then run without chloroquine. In order to resolve the two chiral forms of the trefoil, the first gel dimension should run at low voltage as long as possible. We found that running agarose gels of 40 cm in TBE buffer (89 mM Tris-borate, pH 8.3, 2 mM EDTA), at ≤80 V during ≥70 h (e.g. over the weekend) yields the desired resolution.

In the second gel dimension, a different concentration of chloroquine is included to increase the writhe of the DNA, such that the most negative Lk topoisomers reduce their negative supercoils, and the less negative Lk topoisomers develop positive supercoils (Figure 2D, right). As a result, Lk topoisomers of unknotted and knotted molecules that had similar compaction during the first gel dimension, acquire different velocity during the second (Figure 2D, right). To expose the DNA trefoils in the second dimension, the concentration of chloroquine should be about five times the amount used during the first (i.e. 5–8 μg/ml chloroquine). When the first dimension contained no chloroquine, its concentration in the second should be about 0.6 μg/ml. DNA electrophoresis in the second gel dimension should be run at ≥120 V during 6–10 h, to ensure that linear DNA fragments move ahead circular molecules and do not overlap with the knot signals.

### Test and validation of the method using DNA molecules knotted *in vitro*

**Figure 3.**
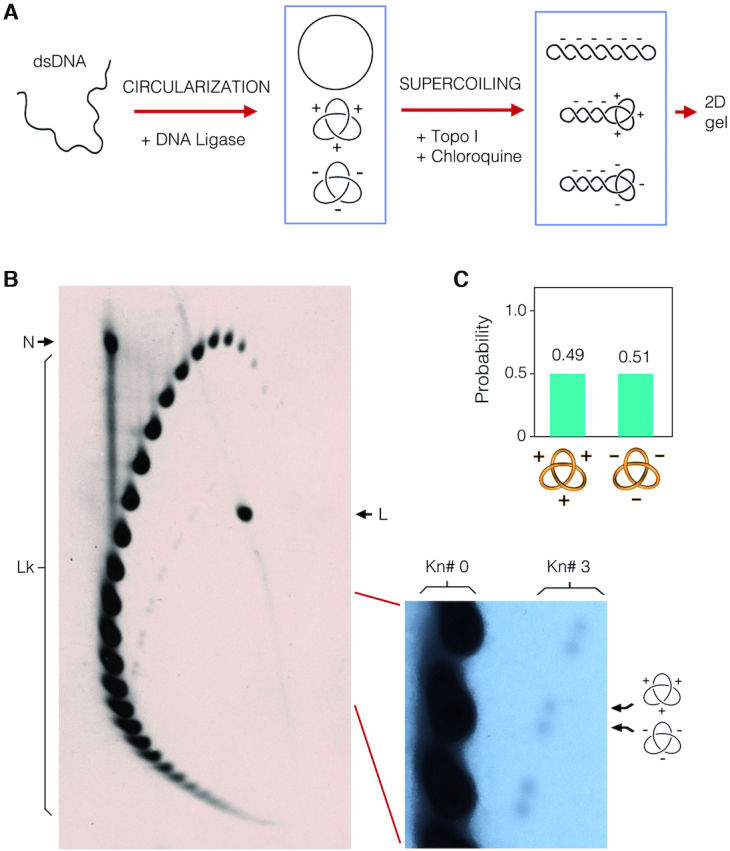
Test of the electrophoresis procedure that discerns DNA knot chirality. (**A**) A linear 4.4-kb DNA fragment was circularized in free solution with T4 DNA ligase to produce a small fraction of molecules containing a trefoil knot. Negative supercoils were subsequently introduced by incubating the circularized DNA with topoisomerase I in presence of 250 μg/ml chloroquine. (**B**) The gel-blot shows the DNA products after high resolution 2D-gel electrophoresis carried out in 0.9% agarose gel (40 × 23 cm) in TBE. The first gel dimension (top to bottom) was run at 80 V for 70 h in TBE (89 mM Tris-borate, pH 8.3, 2 mM EDTA). The second gel dimension (left to right) was run at 120 V for 10 h in TBE containing 0.65 μg/ml of chloroquine. Lk, linking number topoisomers. N, nicked unknotted circles. L, linear DNA. The enlarged gel section shows the signal of Lk topoisomers of unknotted molecules (Kn# 0) and of molecules containing either a positive- or negative-noded trefoil knot (Kn# 3). (**C**) Probability of the two chiral forms of the trefoil knot.

To test the method, we first examined the chirality of knotted DNA molecules produced during the circularization of a linear DNA molecule in free solution. This process yields a small fraction of knotted molecules, mostly trefoil knots (1,6), with equivalent amount of positive- and negative-nodded forms (22). To this end, we circularized a 4.4-kb linear DNA with T4 DNA ligase, introduced negative supercoils by incubating the ligated DNA with topoisomerase I in the presence of 250 μg/ml of chloroquine, and followed our 2D-gel electrophoresis procedure to disclose the chirality of the DNA trefoils formed (Figure 3A). As shown in the gel-blot in Figure 3B, Lk topoisomers of negatively supercoiled molecules were resolved forming an arc of strong signals, whereas Lk topoisomers containing a trefoil knot produced a secondary arc of lower intensity. As predicted, this secondary arc was formed by doublets that corresponded to the two chiral forms of the trefoil. As the signals of each doublet had equal intensity (Figure 3C), this experiment demonstrated equal production of positive- and negative-noded forms during the circularization of free DNA.

**Figure 4.**
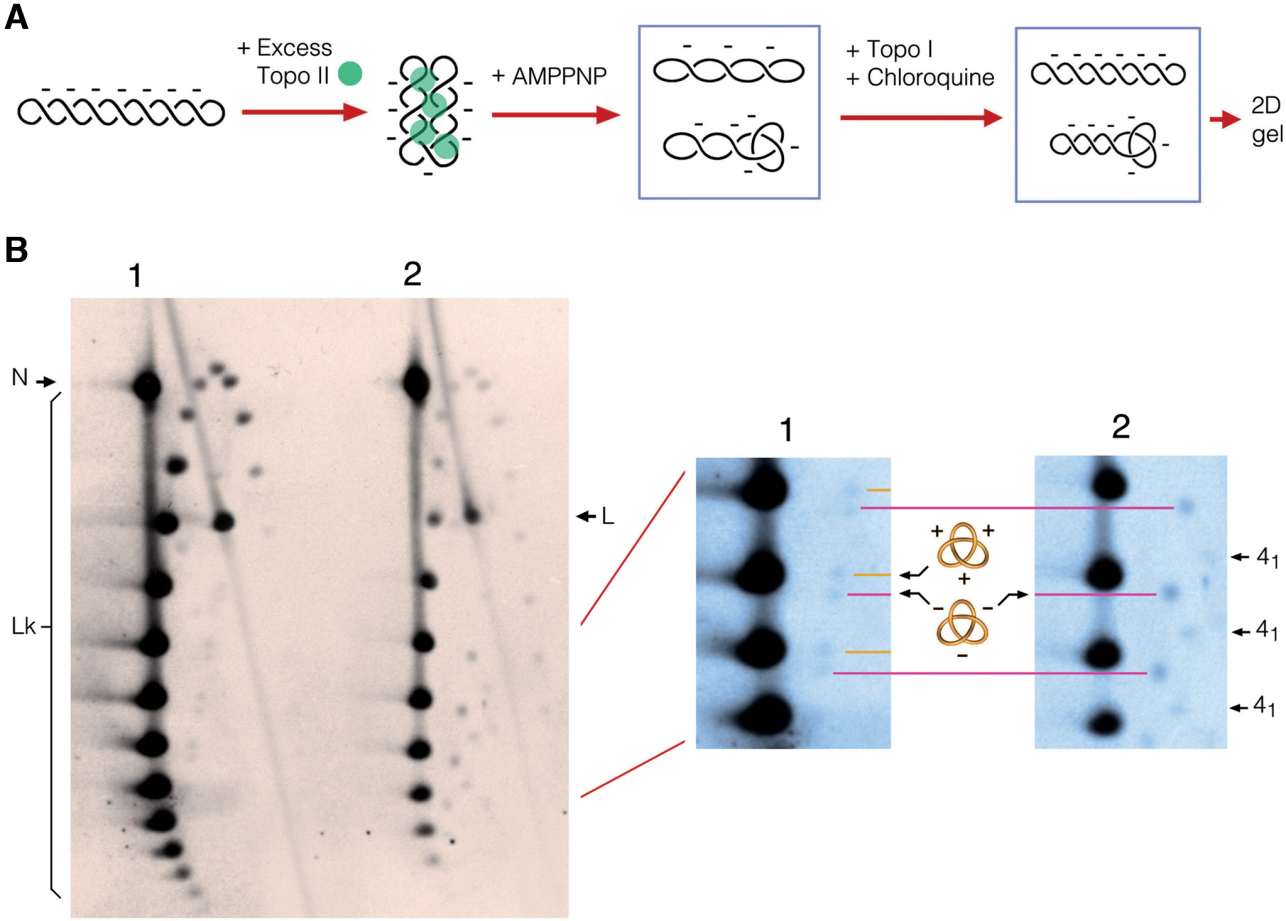
Validation of the relative gel position of positive- and negative-noded trefoil knots. (**A**) A negatively supercoiled 4.4-kb DNA plasmid was incubated with a molar excess of topoisomerase II and AMPPNP to produce essentially negative-noded trefoil knots. Negative supercoiling was subsequently adjusted by incubating the circularized DNA with topoisomerase I in presence of 250 μg/ml chloroquine. (**B**) The 2D-gel electrophoresis compares a sample of DNA circularized in free solution (lane 1) with the products of topo II-mediated knotting of negatively supercoiled DNA described above (lane 2). The first gel dimension (top to bottom) was run at 80 V for 74 h in TBE. The second gel dimension (left to right) was run at 120 V for 6 h in TBE containing 0.65 μg/ml of chloroquine. Lk, linking number topoisomers. N, nicked unknotted circles. L, linear DNA. Enlarged gel sections of lanes 1 and 2 are aligned to show the correspondence in the signals of the trefoil doublets. Additional signals in lane 2, plausibly corresponding to Lk topoisomers of the knot 4_1_, are indicated.

To corroborate that the faster band of each doublet was the negative-noded trefoil, we conducted another experiment. We treated negatively supercoiled DNA with a molar excess of topoisomerase II and AMPPNP, a non-hydrolysable analog of ATP. In this reaction, rather than removing supercoils, the topoisomerase condenses and interlocks plectoneme branches of the supercoiled DNA, producing essentially negative-noded DNA knots (Figure 4A). Next, we adjusted the negative supercoiling of the DNA products by incubating the sample with topoisomerase I in the presence of 250 μg/ml of chloroquine, and electrophoresed it adjacent to a sample of circularized DNA prepared as in Figure 3A. As shown in the gel-blot in Figure 4B, the arcs of Lk topoisomers containing a trefoil knot were visible in both samples. As expected, the negative-noded forms produced in the topo II reaction were revealed as the fastest band of each doublet. In this sample, it was possible to discern also an additional arc of knotted Lk topoisomers, plausibly of the achiral knot of four nodes (4_1_).

### Application of the method in DNA knots formed *in vivo*

**Figure 5.**
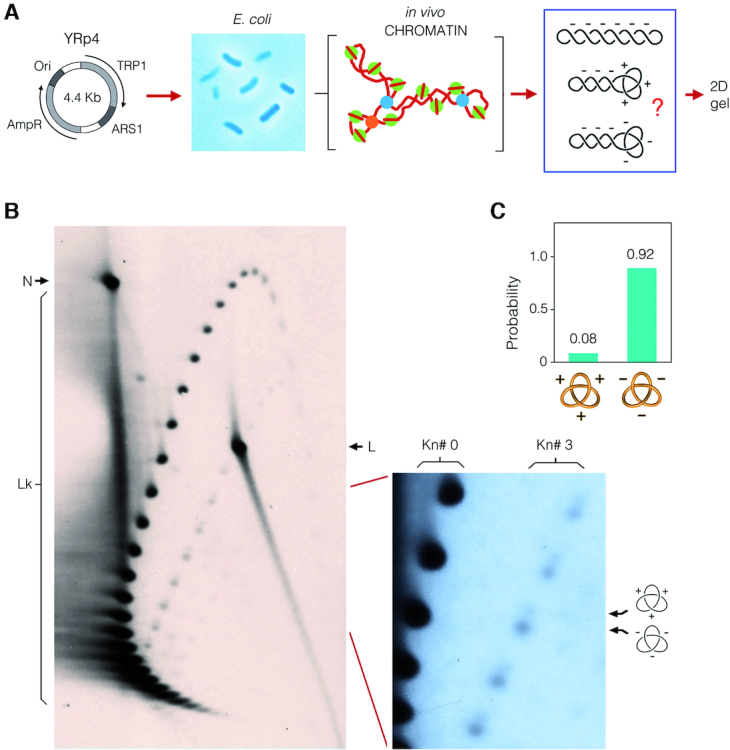
Quantitative disclosure of DNA knot chirality in bacterial chromatin. (**A**) The YRp4 circle was used to transform *Escherichia coli* cells. DNA was extracted from exponential cell cultures using the alkaline lysis method, which captures the topology of DNA *in vivo*. The bacterial DNA sample was examined by 2D-gel electrophoresis. (**B**) The gel-blot shows the in vivo topology of YRp4. The first gel dimension (top to bottom) was run at 80 V for 70 h in TBE containing 1.5 μg/ml of chloroquine. The second gel dimension (left to right) was run at 120 V for 10 h in TBE containing 8 μg/ml of chloroquine. Lk, linking number topoisomers. N, nicked unknotted circles. L, linear DNA. The enlarged gel section shows the signal of Lk topoisomers of unknotted molecules (Kn# 0) and of molecules containing either a positive- or negative-noded trefoil knot (Kn# 3). (**C**) Probability of the two chiral forms of the trefoil knot.

**Figure 6.**
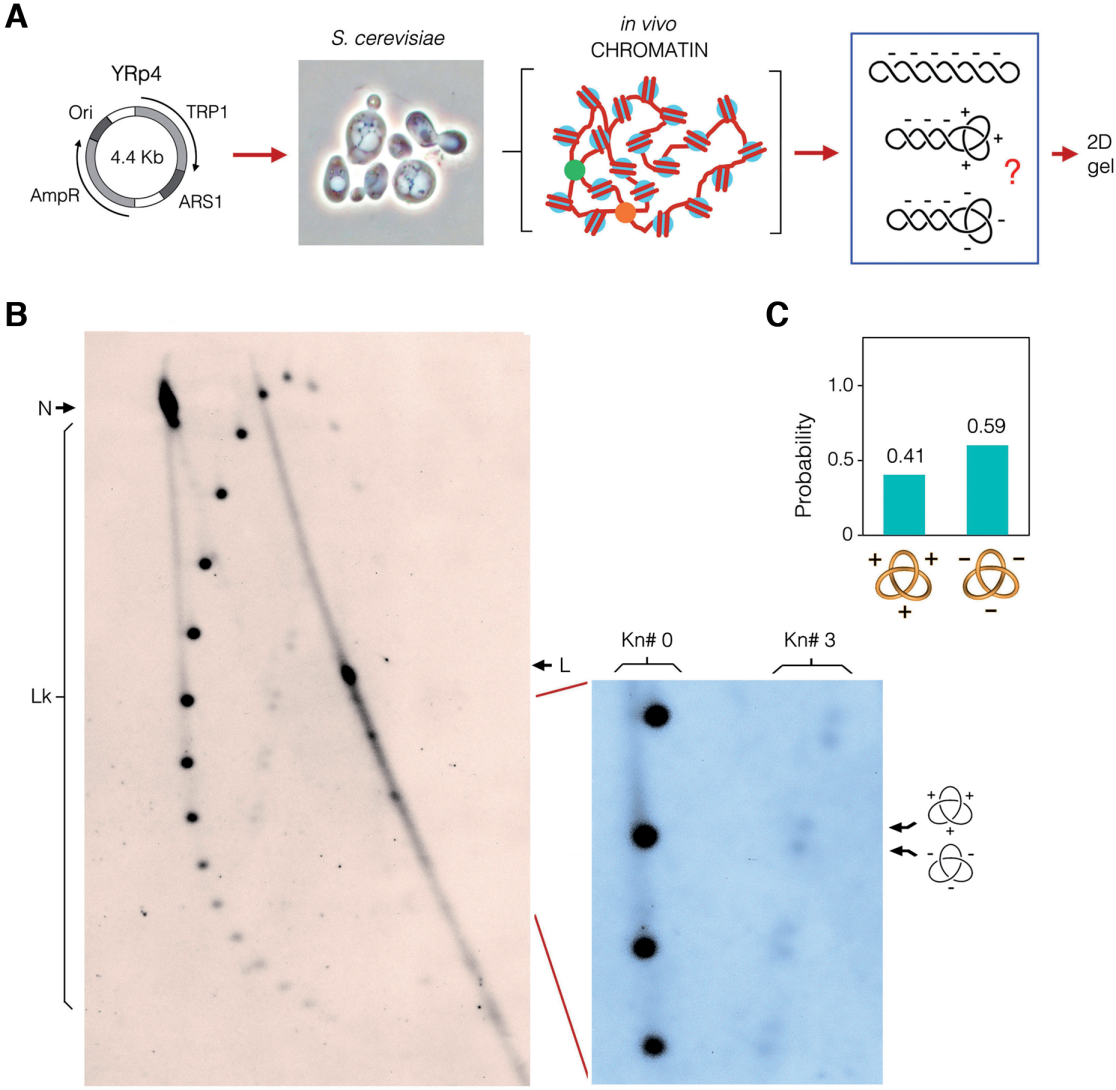
Quantitative disclosure of DNA knot chirality in yeast chromatin. (**A**) The YRp4 circle was used to transform *Saccharomyces cerevisiae* cells. DNA was extracted from exponentially growing cultures after fixing the topology of intracellular DNA. The yeast DNA sample was examined by a 2D-gel electrophoresis. (**B**) The gel-blot shows the topology of YRp4 minichromosome. The first gel dimension (top to bottom) was run at 80 V for 70 h in TBE containing 1 μg/ml of chloroquine. The second gel dimension (left to right) was run at 120 V for 10 h in TBE containing 5 μg/ml of chloroquine. Lk, linking number topoisomers. N, nicked unknotted circles. L, linear DNA. The enlarged gel section shows the signal of Lk topoisomers of unknotted molecules (Kn# 0) and of molecules containing either a positive- or negative-noded trefoil knot (Kn# 3). (**C**) Probability of the two chiral forms of the trefoil knot.

After validating the method, we used it to interrogate the chirality of DNA knots formed in living cells. As shown in previous studies, about 1 to 5% of circular DNA molecules extracted from *E. coli* (9,13) and *S. cerevisiae* (17) cells contain knots (mostly trefoils). However, the chirality of these knots has not been determined or quantitatively analyzed. Therefore, we transformed *E. coli* and *S. cerevisiae* cells with YRp4, a 4.4-kb DNA plasmid that can propagate in both cell systems (17) (Figures 5A and 6A). We extracted the DNA from the bacteria and yeast cultures, conducted the 2D-electrophoresis procedure, and probed the gel-blots with YRp4 sequences. As shown in Figures 5B and 6B, secondary arcs of Lk topoisomers containing a trefoil knot were discernible in the form of doublets in the samples of both *E. coli* and *S. cerevisiae*. In the *E. coli* sample, it was possible to discern also two additional arcs of knotted Lk topoisomers, plausibly corresponding to the knots of four (4_1_) and five nodes (5_1_ and 5_2_) (Figure 5B). The trefoils doublets revealed that 92% of the trefoils were negative-noded in *E. coli* (Figure 5C), whereas the number of positive- and negative-noded trefoils was more balanced (41 versus 59%, respectively) in *S. cerevisiae* (Figure 6C). The *E. coli* results indicated the dominance of negative writhe, which is most likely the result of the unconstrained negative supercoiling that is maintained in bacterial chromatin. In contrast, the results from *S. cerevisiae* revealed the absence of dominant chirality in the spatial path of the chromatinized DNA, in which most negative supercoils are constrained by nucleosomes.

## CONCLUSION

Quantitative disclosure of DNA knot chirality by the electrophoretic method described here expands the possibility to interrogate how chromatin elements and enzymatic activities alter the spatial trajectory of intracellular DNA. Unlike other approaches that inspect the architecture of intracellular chromatin, the analysis of DNA knots is virtually free of experimental artifacts and provides conformational information in short length scales (few kilobases). The main limitation of this method is that, akin to supercoils and catenanes, knots can be only evaluated in circular DNA domains. Therefore, chromatin elements of interest have to be placed in extrachromosomal circles. Immediate applications of this method include the characterization of the chirality of DNA loops associated with DNA folding activities (cohesins, condensins, chromatin remodelers), site-specific elements (centromeres, replication origins, gene promoters and enhancers), DNA recombination processes and epigenetic modifications of chromatin architecture.
